# High-dose chemotherapy and peripheral blood stem cell support in refractory gestational trophoblastic neoplasia

**DOI:** 10.1038/sj.bjc.6602771

**Published:** 2005-09-06

**Authors:** L M El-Helw, M J Seckl, R Haynes, L S Evans, P C Lorigan, J Long, E J Kanfer, E S Newlands, B W Hancock

**Affiliations:** 1 YCR Academic Unit of Clinical Oncology, Weston Park Hospital, Whitham road, Sheffield S10 2SJ, UK; 2 Charing Cross Hospital, Fulham Palace Road, London W6 8RF, UK; 3 Waikato Hospital, Selwyn Street, PO Box 934, Hamilton, New Zealand

**Keywords:** high-dose chemotherapy, gestational trophoblastic neoplasia

## Abstract

We present retrospectively our experience in the use of high-dose chemotherapy and haematopoietic stem cell support (HSCS) for refractory gestational trophoblastic neoplasia (GTN) in the largest series so far reported. In all, 11 patients have been treated at three Trophoblast Centres between 1993 and 2004. The conditioning regimens comprised either Carbop-EC-T (carboplatin, etoposide, cyclophosphamide, paclitaxel and prednisolone) or CEM (carboplatin, etoposide and melphalan) or ICE (ifosfamide, carboplatin, etoposide). Two patients had complete human chorionic gonadotrophin responses, one for 4 and the other for 12 months. Three patients had partial tumour marker responses for 1–2 months. High-dose chemotherapy and HSCS for GTN is still unproven. Further studies are needed, perhaps in high-risk patients who fail their first salvage treatment.

Occasional patients with gestational trophoblastic neoplasia (GTN) will have an incomplete response to conventional chemotherapy or will relapse from remission. There have been several case reports of high-dose chemotherapy (HDC) and haematopoietic stem cell support (HSCS) in patients with refractory GTN (Collins *et al*, 1991; Giacalone *et al*, 1995; Lotz *et al*, 1995; Nagatoshi *et al*, 1996; Chou *et al*, 1997; van Besien *et al*, 1997; Aoki *et al*, 1999; Knox *et al*, 2002). The results have been mixed and no clear prognostic scoring system has been developed to identify which patients may benefit from HDC.

## MATERIALS AND METHODS

In all, 11 patients with refractory or relapsing GTN were treated with HDC and HSCS at three supraregional trophoblast centres (Charing Cross Hospital, London, UK, Weston Park Hospital, Sheffield, UK and Waikato Hospital, New Zealand) between 1993 and 2004. Three conditioning regimens have been used: the Carbo-EC-T regimen (Charing Cross Hospital) included paclitaxel 75 mg m^−2^, etoposide 450 mg m^−2^, carboplatin with AUC 10 on days −7, −5 and −3, cyclophosphamide 60 mg kg^−1^+mesna on days −5 and −3 and prednisolone 30 mg twice daily on days 0–14. The CEM regimen (Weston Park Hospital) included carboplatin (AUC 15), etoposide 100 mg m^−2^ b.d. days −5 to −2 and melephalan 140 mg m^−2^ day −1. The ICE regimen (Waikato Hospital) included ifosfamide 2.5 gm m^−2^+mesna days −6 to −3, carboplatin 500 mg m^−2^ days −6 to −4 and etoposide 600 mg m^−2^ days −6 to −3.

## RESULTS

Six patients had choriocarcinoma (CC), one patient had placental site trophoblastic tumour (PSTT) and one a mixed dimorphic (CC and PSTT) tumour. Gestational trophoblastic neoplasia followed abortions in five patients, term pregnancy in three and complete hydatidiform mole in three. Four patients were initially judged as low and six high risk. The patient with PSTT was widely metastatic from presentation. The median number of prior chemotherapy regimens was 3 (ranges 1–5). Three patients had falling hCG prior to HDC, and eight had plateaued and five had rising hCG.

One patient had a complete hCG response to the CEM regimen lasting 4 months. Another patient had a complete hCG response to two cycles of ICE and remains disease free at 12 months. Both of the latter two patients scored 1 on the Beyer scoring system (Beyer *et al*, 1996), and had falling hCG levels on salvage chemotherapy prior to HDC. Two patients with plateaued hCG levels on prior salvage chemotherapy had a partial hCG response for 4 months (one with Carbop-EC-T and one with CEM). The other patients had progressive disease.

## DISCUSSION

In patients with refractory GTN, HDC and HSCS has been reported in a limited number of studies. Lotz *et al* (1995) treated five patients refractory to standard therapy, with HDC consisting of ifosfamide, carboplatin, and etoposide and HSCS. Two patients had normalisation of their hCG levels for 68 days and 2 months, respectively. Collins *et al* (1991), Giacalone *et al* (1995) and van Besien *et al* (1997) each reported on one patient with refractory GTN who achieved complete response (CR) with HDC. Knox *et al* (2002) reported on a patient with recurrent post-term CC who was treated with an HDC regimen, consisting of cyclophosphamide, etoposide, and carboplatin, and HSCS and had no evidence of disease 23 months post-transplant. On the other hand, Aoki *et al* (1999) reported failure of HDC and HSCS in a patient with refractory PSTT.

Chou *et al* (1997) reported on a 39-year-old woman with metastatic and resistant nongestational ovarian CC who was treated with HDC (carboplatin, etoposide and ifosfamide) and HSCS with surgical resection of two metastatic lung lesions. The patient obtained CR and remained disease free 17 months later. Similarly, Nagatoshi *et al* (1996) reported on a successful outcome with similar treatment for an adolescent male patient with retroperitoneal pure CC and multiple brain metastases.

In our series, which although retrospective is the largest so far reported, 11 patients were treated for refractory GTN with HDC and HSCS. Most of these cases had suffered multiple relapses and/or resistance to treatment despite the use of the standard first-line chemotherapies as well as a variety of intensive salvage treatments. Most patients (70%) had initially been assessed as high risk. Either Carbop-EC-T (carboplatin, etoposide, cyclophosphamide, paclitaxel, prednisolone) or the CEM regimen (carboplatin, etoposide and melphalan) or the ICE regimen (ifosfamide, carboplatin, etoposide) was used.

Two patients had complete hCG responses; one had originally had hydatidiform mole, stratified as low risk and received five different chemotherapy regimens for disease recurrence with lung and brain metastases. She also had hysterectomy and pulmonary and cerebral metastatectomy followed by an ifosfamide/taxol regimen with complete hCG response. This marker remission was consolidated with HDC and HSCS. She remained disease free with hCG CR for 4 months following transplant. The other patient had originally had hydatidiform mole, stratified as low risk and received four different chemotherapy regimens, as well as having pulmonary and cerebral metastatectomy for recurrent metastatic disease. She received a tandem transplant with the ICE regimen with complete hCG response and was still alive and disease free 12 months post-transplant. The two patients who had complete hCG response each scored 1 on the Beyer system. Three other patients in our study showed 1–2 months partial hCG response to HDC and HSCS; one of them had initially a persistent hydatidiform mole, judged as low risk; the other two patients had CC and were categorised as high risk at initial diagnosis. All three patients each received three different chemotherapy regimens prior to HDC with Carbop-EC-T. All patients who showed a response to HDC and HSCS had had an hCG response to the salvage regimen prior to HDC, which might reflect the chemosensitivity of their disease and could be a clue for proceeding to HDC.

In conclusion, HDC and HSC support for GTN is still investigational and reported in a small number of cases. The cases reported here suggest that, while HDC may be an option, further studies are needed, perhaps in patients with high-risk GTN who fail their first salvage treatment for recurrent disease.
